# How does housing affect end-of-life care and bereavement in low-income communities? A qualitative study of the experiences of bereaved individuals and service providers in the United Kingdom

**DOI:** 10.1177/26323524221110248

**Published:** 2022-07-07

**Authors:** Lorraine Hansford, Felicity Thomas, Katrina Wyatt

**Affiliations:** Wellcome Centre for Cultures and Environments of Health, University of Exeter, Exeter, UK; Wellcome Centre for Cultures and Environments of Health, University of Exeter, Queen’s Drive, Exeter, EX4 4QH, UK; Wellcome Centre for Cultures and Environments of Health, University of Exeter, Queen’s Drive, Exeter, EX4 4QH, UK

**Keywords:** bereavement, end-of-life care, housing, palliative care, public health approaches, socio-economic status

## Abstract

**Background:**

Access to affordable, appropriate housing is one of the key social determinants of health, affecting well-being across the lifecourse. However, beyond a recognition that housing quality is linked to place of death, little is known about the ways in which housing status impacts social, emotional, and practical aspects of dying and bereavement.

**Method:**

The Checking Out project is a qualitative study aiming to explore the ways in which socio-economic status impacts people’s experiences of, and attitudes towards, death, dying, and bereavement in the United Kingdom. Qualitative interviews were carried out with 14 bereaved individuals with experience of poverty at end of life or in bereavement, and 15 professionals supporting individuals in low-income communities. Interviews were conducted via phone/video call, and data include experiences of end of life and bereavement both before and during the pandemic. Transcripts were examined using thematic analysis.

**Results:**

Housing emerged as an important factor affecting people’s experiences, with 7 of the 14 bereaved individuals and all except 1 of the professionals discussing housing-related issues. Participants described ways in which unsuitable housing and housing insecurity impacted practical aspects of dying but also emotional and social well-being at end of life. Housing-related issues affected both patients and their families, though families found it difficult to air these concerns when their relative was dying.

**Conclusion:**

The paper demonstrates how trusted professionals are able to advocate or address the issues faced by bereaved individuals and suggests implications for policy and practice. A greater awareness of the potential impact of housing status across public services, including healthcare practitioners, welfare support, and housing providers, could better support patients and practitioners to address these issues proactively. Housing providers and policy-makers should be included as key partners in collaborative public health approaches to palliative care.

## Introduction

Housing is recognised as one of the key social determinants of health, and evidence that housing status shapes and reinforces health inequalities has been growing over the last 10 years.^1^ It seems logical to assume that as housing impacts on health throughout the lifecourse, it will also impact experiences of dying and bereavement. It is perhaps surprising then that there is little discussion about the role housing might play in palliative care discourse.^2^

Issues surrounding housing inequalities for those at end of life are pertinent, given the plentiful discussion in the recent British media about the crises both in housing and in social care,^3^ and increasing levels of poverty.^1^ However, public discourse and recent policy debates have largely focused on the rights and wrongs of elderly homeowners selling their property to fund residential care,^4^ reinforcing the intersection between inequalities in housing status and inequalities in access to care. Of course, housing, end of life care and bereavement are not just issues that affect the elderly, particularly for those living on a low-income. As well as insecure or substandard housing being linked generally to poorer health,^5^ the risk of ‘avoidable mortality’ is three times higher for those living in the most deprived areas^6^ and the life expectancy gap between the most and least deprived areas can be up to 20 years in England.^7^ Those most likely to experience multi-morbidity and advanced disease are also those least likely to have the resources to make choices about their care.^8^

Interest has grown in recent decades in developing public health approaches to palliative care, partly as a potentially sustainable response to pressure on services to meet increasing needs.^9^ While there is no single term or definition for a public health or health-promoting approach to palliative care, there is an underlying ethos that responsibility for caring for the dying and bereaved should be shared by communities and healthcare professionals.^10^ The range of practices that have been adopted include mobilising community volunteers to provide support and awareness-raising activities focused on preparing for end of life.^9^ Early evidence shows that the development of such initiatives seems most effective in neighbourhoods where there is a tradition of volunteering or community activism,^11^ and while some low-income areas have active community networks, research has shown that, in general, areas with higher levels of poverty have a lack of relationships both within their communities and with local services such as housing and healthcare.^12^ In addition, little is known about the effectiveness of public health approaches in areas with more transient populations, or for individuals who do not feel established within a local social network.

There is, of course, some discussion in the palliative care literature on housing-related issues, particularly in relation to place of death. We know that in general most people in the United Kingdom say they would prefer to die at home^13^ and that dying in hospital is more likely to involve excessive medical intervention, increased suffering, and the exclusion of contributions from friends and family.^11^ We also know that there are links between housing quality and place of death,^14^ with people on a lower income being less likely to feel they had sufficient support to care for someone dying at home and less likely to die at home rather than in a hospital.^15^ There is some research identifying practical barriers to a home death, for example, lack of space for specialist equipment.^16^ However little is known specifically about the views of both people experiencing poverty at end of life and those supporting them about the ways in which their housing situation affects not only their preferred place of death but more generally their experiences of dying and bereavement, and what is important to them at end of life.^17^

End-of-life care policy^18^ and ‘death awareness’ discourse^19^ are dominated by narratives of choice and responsibility and are based on the evidence that the majority of people prefer to die at home. However, a recent Marie Curie report reviewing the barriers to good end-of-life care points out that basing policy on this presumed choice is problematic because it reflects the opinions of the general population and excludes certain population groups, including those with a low socio-economic status, for whom dying at home may not be their preferred choice, may not be possible, appropriate, or safe, and for whom options for place of care may be limited.^20^ The report argues that to ensure that everyone has the best end-of-life experience possible for them we must ‘try to address the reasons why place of death may not be the quality marker it’s believed to be’. Understanding the ways in which contextual factors shape people’s attitudes towards death and dying is therefore vital.

At the most adverse end of the scale, people who are homeless are much less likely than other population groups to access palliative care.^21^ Research shows that obstacles to accessing care extend beyond environmental issues (e.g. lack of suitable accommodation in which to receive care) and include factors such as previous experiences of accessing services and feelings of shame.^22^ Researching barriers to accessing healthcare at end of life, Stajduhar *et al.*^23^ found that people experiencing structural vulnerabilities were so ‘busy living in the moment and surviving’ that advance care planning, provision of palliative care, or discussions about death and dying were ‘simply absent from participants everyday lives’ and that an awareness of the palliative care services potentially available was minimal both for individuals themselves and the community-based service providers they were in contact with.

In their review of the literature addressing poverty at end of life, Rowley *et al.*^17^ acknowledge the lack of research drawing on lived experience and question whether researchers and clinicians in this field actually know whether they are addressing the issues that are important to the people experiencing these inequalities. This project, Checking Out, has been co-created with people living in low-income communities who have experienced bereavement and with service providers from two hospices. The study involved interviews with bereaved individuals with experience of poverty at the time of their relative or friend’s death, and professionals involved in the field of death and dying who support people in low-income communities, to gather insight into their experiences and understand the impact of structural as well as economic inequalities on their experiences. The broader range of issues that emerged will be presented elsewhere; however, issues related to housing and their impact on the person dying as well as those caring for them were a common recurrence within the data. Bowlby^3^ argues that housing has increasingly become not just a physical space but a ‘resource for care’, as ‘an asset to finance care, as a built form, as a source of identity and ontological security, and as a base for fostering networks of support’. Understanding housing as a ‘resource for care’ in this broad sense is vital if we wish to fully comprehend barriers to dying and grieving well. The data from this study illustrates some of the ways in which housing impacts on multiple aspects of dying; not only environmental conditions relating to space and place, but also social, emotional, and psychological aspects of death, both for the dying and the bereaved.

## Methods

Checking Out is a qualitative study which aims to explore the notion of ‘a good death’ within low-income communities and to understand the impact of poverty on experiences of end of life and bereavement. The original study design (beginning October 2019) included two strands: community engagement activities in settings such as community cafes to inform the research questions, followed by focus groups and interviews. Engagement activities were interrupted by the COVID pandemic in March 2020, and after discussion with the advisory group, community groups and individuals who had already shown an interest, the design was adapted so that data could be collected remotely. This paper reports on the findings from interviews (*n* = 29) carried out from July 2020 to March 2021 with participants coming from different areas in the United Kingdom including England, Wales, and Scotland.

Interviews were conducted with 15 professionals who self-identified as having extensive experience of supporting people living on a very low income at end of life or in bereavement, either due to their geographical location (e.g. funeral directors situated in areas scoring highly in the Indices of Multiple Deprivation) or due to the nature of their role (e.g. providing welfare or debt advice to families experiencing poverty at end of life or in bereavement). Participants were recruited using convenience sampling and snowballing methods, for example, publicising the research through the National Bereavement Alliance and Association of Palliative Care Social Workers, and directly contacting relevant professionals who had expressed an interest during the community engagement phase. Professionals had a variety of roles including funeral directors, faith leaders, welfare advice workers employed by charities or hospices, palliative care social workers, and a credit union employee who worked closely with a funeral director to support families experiencing funeral poverty (see Table 1).

**Table 1.** Characteristics of interview participants.

Participant characteristic	Bereaved individuals	Professionals
Total		
	14	15
Age range		
18–34	3	1
35–54	5	11
55+	6	3
Gender		
Female	10	11
Male	4	4
Other	0	0
Ethnicity (self-defined)		
Bangladeshi	0	1
Other	0	1
White British	13	13
White European	1	0
Professional role		
Charity/specialist advice worker		3
Cleric/celebrant		3
Credit union relationship manager		1
Funeral director		5
Hospice/health charity welfare rights advisor		2
Palliative care social worker		1

Fourteen interviews were held with bereaved individuals who were also recruited using convenience sampling and snowballing methods; some were informed about the study and invited to take part by one of the professionals interviewed with whom they had an existing relationship, and some expressed an interest in participating during early engagement work. Participants either chose to contact the researcher directly or gave permission to pass on their contact details. Individuals were eligible to take part if they were 18 or older and had been bereaved within the last 5 years. During an initial conversation, the researcher explained that the research focused on poverty at end of life, and individuals self-identified if they felt their experience was relevant. In terms of their relationship to the deceased, four participants discussed the death of a parent, four a spouse/partner, one an ex-spouse/co-parent, one a sibling, one an adult child, one a friend/housemate, and two discussed multiple bereavements including close family members.

Interviews were conducted either by telephone or video call (according to participant choice), with participants giving informed verbal consent. The duration varied from 25 to 69 minutes for bereaved individuals and 47–154 minutes for professionals. The semi-structured interviews were guided by a topic list which included the question ‘In what ways do you think worries or insecurities around things like money, housing, jobs, debt etc can impact on people going through this kind of situation?’ (i.e. end of life and bereavement). Seven of the 14 bereaved individuals and all except one of the professionals raised issues related to housing during the interview.

Interviews were audio recorded and transcribed verbatim, and all participants were asked if they would like the anonymised transcripts to be returned to them for comment and/or correction. Seventeen said yes and two participants added an extra written comment via email in response, which was added to the transcript. The characteristics of the participants are presented in Table 1.

Following the principles of thematic analysis^24^ interview data were coded and analysed using QSR International’s NVivo 11 Software. This involved iterative thematic coding, combining both a deductive approach informed by the topic guide and an inductive approach, whereby codes were derived from the data; all codes were then integrated into a coding framework. Feedback on the formulation of themes and sub-themes was sought from the project advisory group who had access to the anonymised data.

## Results

### Choice about where to die

As expected, both professionals and individuals talked about housing-related factors determining whether people approaching end of life were able to make a genuine choice about where they would like to die. Prohibiting factors for dying at home included whether there was space for a hospital bed or specialist equipment and whether extra costs could be met (such as heating, medical supplies, transport for the patient and carers). As one individual explained, though supplies would in theory be provided by the National Health Service, this was not something they were aware of until a late stage, and she relied on her daughter’s savings to meet the costs of caring for her father at home

I had no money at all. I was having to go back and forth, [daughter] was having to go back and forth . . . bought bus cards and things like that. If she didn’t have her savings, we were up the creek because the only way we managed to live and buy everything we needed . . . Because before he passed he became incontinent, so we were having to buy [incontinence pads] . . . About a week before he passed away we found that the district nurse should have been supplying them, whereas we had been buying them.

Professionals reported that the cost and time to make necessary adaptations to a home could be an obstacle, as even if people were entitled to financial help from the state, the timescale would be impractical. For people living alone, whether there was space for carers to stay (family or paid), and whether this space was deemed safe for healthcare professionals to visit, was another factor. One participant reported that their neighbour, whose husband was receiving end-of-life care in hospital, was worried about whether family members would be able to visit him before he died, as they did not live nearby, and she did not have space for them to stay.

Professionals stated that being homeless or temporarily housed ‘takes options away’. One hospice Welfare Rights Adviser described how she had ‘fought for the rights’ of a man with a palliative diagnosis who did not want to die in hospital where he ‘didn’t react well’, but had previously been sofa-surfing, with no permanent home. She described how he was initially

discharged from hospital [. . .] to a bedsit that had water coming through the roof and a shared toilet and a shared kitchen with multiple occupants that he didn’t know.

As a result of her advocacy, he was eventually allocated a suitable flat which the hospice furnished and where they supported him to die. She observed, however, that many homeless people are not referred to hospices so would not receive this care. In situations where a patient’s social housing was considered unsuitable for palliative care, professionals reported that it was very difficult to sort out alternative housing due to the lack of housing stock and lengthy bureaucratic processes. One Welfare Rights Adviser echoed others when reporting that ‘sometimes the person has died before we’ve managed to get somewhere for them’.

Obstacles to a home death extended beyond the practical or financial. Some professionals had observed that people in inadequate housing could feel a sense of shame or hesitancy about strangers coming into the home. One individual, who cared for their father at the end of his life, described how they felt that their lack of privacy was worsened by housing association wardens ‘constantly at the door’ with concerns about fire safety (because the father was bed bound in a 12th floor flat), which they were unable to address. She described how she felt this lack of privacy and sensitivity continued after her father’s death

We are all there waiting for the undertaker to come and there’s only one lift to the floor . . . So they took the back panel out and stuck a big note on the door – ‘Do not use, undertaker coming’. Just put ‘out of use’ or whatever. But no, they stuck a flippin’ great notice on there.

### Isolation

Advice workers described examples when some individuals who ‘chose’ to stay in unsuitable housing experienced a lack of mobility and greater isolation as a result, when ‘in the last few months of their life, they can’t even get out of their flat’. Feeling constrained by the housing system left advice workers feeling frustrated and limited in their ability to help

I really, really hate seeing people who are completely isolated in the last few months of their life because we can’t sort out housing for them, and [they] are not able to go out. I’ve just got so many people in my mind that I feel were really let down with their housing situation, and perhaps then dying in hospital which is what they didn’t want, because their housing meant that they couldn’t die at home. That to me is a massive thing, is the housing issue.

The link between housing and isolation at end of life was not limited to accessibility. Even when people were allocated suitable housing at end of life, this was often in a new area away from established support networks. As one Welfare Rights Adviser commented

I’ve found this for the last 25 years of community work – those people who have got that extra support of family and friends, they do seem to have a better experience . . . the wider your friendship and community is, the more you get told ‘There’s this you can do . . . have you applied for this?’ . . . Whereas somebody who’s quite isolated doesn’t perhaps find out about that.

### Cumulative inequality

Professionals also gave examples of people who were isolated at end of life not necessarily because of their illness, but by other circumstances that had forced them to move away from their support network, such as homelessness or escaping domestic violence. The data indicate that quality of care at end of life can be a result of inequalities experienced across the lifecourse. One faith leader, for example, compared a middle-class community that he had previously worked in, where the nearby hospice was well-known within the community and local people were involved in volunteering and fundraising, with the low-income community in which he was currently working, where people were much less likely to be aware of the hospice or know people who had died there; he observed that accessibility was not just about geographical distance but expectations in relation to care and support

I think accessing care like Macmillan Nursing and things [. . .] I think people are more hesitant to ask for it. And I think some of that is a worry that it’s going to cost them, that if they sign up to say they want Macmillan care or similar or hospice care, they’re going to be faced with a bill for hundreds or thousands of pounds. And so, I guess some of it is then about just making sure people have got clear information about services that are going to be free, and that they can access, and they’ve got a right to access.

Another practitioner described projects she had developed to promote advance care planning among underserved communities. Working with a homeless charity, her experience was that homeless people were unlikely to engage not only because their immediate concerns (e.g. food and shelter) had not been addressed, but because they did not trust that authorities or services would listen to their preferences

I guess their thoughts were ‘Well these issues aren’t being sorted for me now, so why should I trust that anyone is going to listen to what I want in the future?’

This practitioner had found that similarly some people within minority ethnic communities expressed the view that they did not feel listened to by health professionals now, so the concept of advance care planning felt pointless – demonstrating that messages about the importance of planning and choice may seem irrelevant to communities who do not feel that their voice is heard.

### Worries about those being left behind

Both professionals and individuals reported that concerns about housing insecurity after a death were common, both for the patient and their families. Although this issue may not seem directly related to palliative care, it is important not just because of the distress it causes for the bereaved but because the anxiety invoked had a significant impact in the period of time before death. As one adviser commented, ‘Its difficult to concentrate on this notion of a good death if you’re worrying about the rent and the mortgage’.

Concerns related to a range of issues, including the security of bereaved family members who may be currently living with the patient but were not on a tenancy agreement. One adviser gave an example of a patient who lived with two non-dependents in social housing and was worried that they would become homeless upon her death. The adviser worked with the housing association to add the family members to the tenancy agreement, alleviating her concerns. Conversely, a celebrant gave an example of a situation in which there was no intervention

The person who was caring for them – their son or daughter – gave up their work and their own home to move in and be a full time carer for their parent, but because they weren’t on the tenancy agreement, as soon as that person died they forfeited all rights to be there and were literally given notice to leave within a matter of weeks. They not only lost their own home, their own job, their own sense of identity and their relative, but they also then lose their home as well.

There were also examples of bereaved individuals who were worried about being able to pay the rent after someone dies. This could be in social housing because they were aware that the ‘bedroom tax’ would become applicable, or in private rented accommodation which they could not afford to pay for alone. Bereaved individuals described how the stress of their housing situation interacted with their grief

They were charging me for bedroom tax . . . I’m in a three bedroomed house and they want to charge me for the two bedrooms I’m not using now . . . we’ve lived in this property 16 years, so we were married 36 years so we’ve got a lot of memories in this house . . . it [moving] is going to be upsetting.

One person described being given 3 weeks to leave their privately rented accommodation after their partner died and having to deal with the deceased’s belongings as well as their own grief, and the worry of their housing situation, while future tenants were being shown around the property

The biggest deal was finding somewhere to live when you have three weeks to find somewhere . . . I was going stir crazy . . . I was driving everywhere, I tried every estate agent you could think of. I thought I just want to be with my little dog, If not, what am I going to do? I am going to have to bloody try and sleep in the car or something . . . If it was in the summer it might be a bit easier because I suppose I could buy a tent and live in that.

Professionals recounted numerous examples of relatives becoming homeless after a bereavement, causing a mental as well as practical strain for families. They recognised that although this was often a ‘massive concern’ for both patients and their relatives, family members often found it hard to talk about while the patient was alive and felt guilty about being ‘selfish’ if they aired their worries.

### Constraints to grieving

The regulations imposed by landlords after someone died affected bereaved people who had not lived with the deceased person, as well as those who had been co-habiting. In numerous examples, individuals cited their experiences of having to clear out a rented property after a death as traumatic. It was not uncommon for people to be given a short time period to do this by a landlord (whether private or social housing); in one case, this was less than a week. Other individuals talked about their worries about having to pay rent while the property was not cleared, yet (in one example) not wanting to visit the property after a traumatic death

I was worried about my daughter’s flat because rent would need to be paid on the flat and I was thinking I can’t cope with clearing out the flat. I thought I don’t think I can go in there yet and I was thinking what am I going to do? And then not wanting to dispose of all of her things but not feeling able to go through them and thinking about paying for storage or where on earth am I going to put these things? It just adds to everything else. Makes it all a bit harder to cope.

Some individuals had struggled with the costs of transport to move and store belongings, and mentioned being threatened with court, or the loss of their own tenancy, if a property was not cleared within a short timeframe. As a result, some people felt forced to deal with their relative’s possessions before they were ready, or to dispose of them due to the cost of storage

Most of it will end up in a skip and it is a shame because that is somebody’s life you are having to throw away. Whereas if you had storage space or some place to put it, you wouldn’t throw half of it away, because that is memories.

Comments from professionals about people being ‘almost unable to grieve because they are worried about losing their home’ were typical, as they observed the trauma that individuals experienced when coping with a bereavement combined with the loss of their home. Speaking about one woman who could no longer afford to live in the home she had shared with her disabled husband for 10 years one professional commented

she’s got no transport, she’s going to lose her home, she’s lost her husband, and then her mental health is rock bottom. We are supporting her with her mental health and we’ve referred her to a local mental health team as well, but that’s still not going to help her financially.

## What might help?

Examples in the data in which people had received effective support to address housing-related stresses at end of life appeared to share some common elements.

### Having an advocate

A number of individuals talked about receiving help or advocacy from somebody during illness or bereavement (in relation to housing or financial insecurity) and how valuable this had been at a time when they had felt extremely stressed or vulnerable. Those working in a supportive role stressed the tenacity that was needed to achieve positive outcomes, using language such as ‘fighting for’, ‘badgering’, or ‘putting pressure on’. As one adviser observed

If the end is in sight, do you have the energy to advocate for yourself? I doubt it.

As well as an advocate being able to commit time to ‘fighting’ for accommodation, advisers were also able to build relationships with housing providers over time and had acquired knowledge and contacts to negotiate the systems. However, there were also examples of advocates who did not necessarily have any specialist knowledge but were willing to step in when the person had no other support, for example a celebrant who had liaised with the council to advocate for a bereaved person overwhelmed with anxiety about their tenancy agreement. One individual talked about how difficult it might be to know how to access help, particularly at a stressful time

there are people out there that know; they’ll tell you, ‘Oh, do this, do that’. Yes, Citizens Advice and things like that, but not everybody does that, and they need to. Because when you lose somebody, you just don’t think straight anyway, and you just need somebody outside the family to help.

### Trusted relationships

The data showed clearly that key to successful advocacy was not just professional knowledge but the quality of a relationship. One individual described being in temporary accommodation in a caravan park after his partner died. He initially went into a local ‘community hub’ only to use the laundry facilities, but while chatting disclosed that he did not have a fridge, which the hub was then able to provide. He described getting to know a volunteer there who is now helping him approach the council to seek secure accommodation

It is something [name] mentioned at the hub. This is only a temporary thing and of course I have to move from here because it is a holiday let . . . she is helping me try to get into that system and she’s been in touch with my GP.

One adviser described a situation she had encountered when managing a foodbank. Visiting a bereaved client living in social housing, a worker had discovered that the client was hoarding and that her living conditions were ‘almost like living rough but in a house’. The adviser’s experience of working in low-income communities meant that she was aware of the perceptions of ‘services’ that can prevent people seeking help

Folk are very scared of authorities, because folk who live in poverty worry that they’re either going to lose their house or they’re going to lose their kids. So, if you try and give them help in the shape of a housing officer, there’s a huge fear there. And in the same way around social work.

The adviser explained that the housing team had ‘thought she was fine’ and that the situation only came to light because foodbank staff had built a relationship and were trusted to come into her home

the trust that this person, this particular client, had in my team member was immense, because she was so filled with shame as to the situation she was living in, and so fearful that she was going to lose her house.

This wariness of services means that although people might need someone to ‘fight’ for them at certain times in their lives, as described as above, those able to advocate also need to understand how difficult it might be to ask for or accept help

sometimes you just have to be that person’s voice until they find their own voice, to be able to bat for them a little bit. And also, to maybe express the fact that they’re human and they’re scared. And sometimes when people, when the scared bit of our humanness arises, it doesn’t always look like scared.

Professionals also mentioned the importance of continuity over time for building relationships where people feel able to seek support. One hospice advice worker explained how their welfare team provide advocacy for families beyond the initial bereavement period

One lady last week, her husband had passed away and she needed to move into a smaller property. Again, we liaised with the housing associations to get that done for her. So we continue that relationship afterwards, it doesn’t end when the patient dies. We continue to support them as long as they need it.

Trusted relationships were here facilitated by both the practitioner’s attitude and their sustained availability over time. One celebrant suggested a ‘One Stop Shop’ providing drop-in advice, information and support about any aspect of dying and bereavement, and located within low-income neighbourhoods, as an ideal model. Another funeral director described their aspiration for their premises to operate in a similar way as a community resource.

### Home visits

Having had to adapt their working practices due to COVID-19, several professionals noted that home visits were vital to properly identify and understand needs. Examples cited include the hoarder described above who had been able to hide her situation from housing workers, a woman with a palliative diagnosis who had been re-housed after escaping domestic abuse whom, when visiting, the adviser discovered had no beds for herself or her children, and a patient living with lung cancer in a third-floor flat who was unable to use the stairs. As one adviser explained, ‘if you’re trying to work on a telephone you don’t see those problems’. In a post-pandemic context in which many services have recognised some benefits and efficiencies to offering remote support, it is important to flag how this may exacerbate existing inequalities in access to support.

### Rethinking narratives of choice

Some professionals experienced incongruence between the narrative of choice that tends to be prevalent in notions of a ‘good death’, and the realities experienced by the people they were supporting. One adviser explained that although hospice nurses will discuss and try to support patient’s wishes, they must also recognise constraints. For example, the patient might not be able to die at home if ‘the set up at your property doesn’t allow that to happen’, or in the hospice if no bed is available. This adviser saw her role as to facilitate a dignified death, in whatever sense the patient saw this, and interestingly again recognised that they may need to ‘fight’ for this

everybody who comes to us and they’re dying, we will support them in any way we can for them to die a really dignified death . . . we will fight for the rights for the person and how they want to die.

One individual described how difficult she found it to respond to her father’s request to care for him at home and how much she appreciated the support of hospital and hospice doctors in making decisions about what was realistically possible both for herself and her father

He was desperate to go home . . . I was pretty much like ‘I can do it. He can come home. We can set something up in the living room because he can’t get up the stairs’. Lots of impossible things. They were really good at handling me and also him as well. They were like ‘[Going home] isn’t possible’ . . . [his consultant] was amazing . . . He would continually ask how I was feeling about the situation . . . The care they gave was really straight up and practical’.

Effective support in preparing for death therefore appeared to be about the careful balancing of choice and pragmatic possibilities with a detailed understanding and consideration of the individual, their family and their home context.

## Discussion

The findings show that stable and suitable housing is a key factor influencing people’s experiences of dying and bereavement; not just as a financial and environmental resource, but as Bowlby argues as a source of identity, security, and a base for fostering support networks.

In her research investigating barriers to accessing end-of-life care for structurally vulnerable populations, Stadjuhar *et al.*^23^ found that healthcare providers did not always understand how concerns about everyday requirements such as housing and food security might influence a patient’s ability to access services and that it was only when issues were addressed as an essential component of palliative care that participants were more likely to obtain quality care. The examples within our data suggest that it is important not only for those working with low-income communities to understand the structural realities of their everyday lives, but how their previous experiences may mean that they are fearful of the involvement of services and need time to build trust.

Some of the findings confirm those reported in other research, particularly in bereavement: Corden and Hirst^25^ state that one of the most pressing financial issues for bereaved partners is ‘how safe’ their home is and that housing insecurity can negatively impact the grieving process. In their scoping review of social and structural inequity following expected death, Bindley *et al.*^26^ found that studies highlighted unequal social status in bereavement related to gender, class, sexuality, ethnicity, and age and that associated outcomes included housing insecurity. They assert that the intersection of social and structural inequities contributes to ‘layered and patterned experiences of disadvantage’, chiming with our finding that attitudes towards preparing for death and accessing care are influenced by myriad experiences of accessing care or services across the lifecourse. Listening to people’s experiences of bereavement is important when considering how to improve access to palliative care – not just to inform support for carers, but because experiences of bereavement shape people’s attitudes, expectations, and fears in relation to their own death.

The recent Lancet Report on the ‘value of death’ argues for a ‘realistic utopia’, one principle of which is to tackle the social determinants of death, dying, and grieving.^11^ The report recognises that improving experiences of dying requires change within the wider and complex ‘death system’ – an argument that builds on the growing momentum for developing public health approaches to palliative care.^27^ Our data show the importance of considering housing providers as part of the death system. Any attempt at building ‘compassionate communities’10, in which people feel supported when approaching end of life, needs to consider not only individual need in relation to accessible and secure housing, but how housing policies and systems can address structural inequity at end of life.

### Implications for policy and practice

The Report of the All-Party Parliamentary Group for Terminal Illness inquiry includes 20 recommendations for government, local authorities, energy providers, and health and social care professionals to address unsuitable housing and unaffordable housing and energy costs at end of life.^28^ They note that the National Institute for Clinical Excellence^29^ has issued guidelines to health and well-being boards in England and Wales which include the commissioning of local single-point-of-contact health and housing referral services to support vulnerable people in cold homes and provide tailored solutions, but that implementation is patchy. However, if universally available, an integrated system such as this could have huge potential for identifying and addressing not only cold homes, but the other housing-related issues that impact both directly and indirectly on well-being at end of life.

Marie Curie have led the way in terms of research and campaigns highlighting the ways in which unsuitable housing and fuel poverty contribute to inequalities at end of life.^20^ A report in 2014 looking at the experiences of social housing providers in Wales in facilitating high-quality end-of-life care for their tenants found that most housing providers had not, as an organisation, considered end of life, but were positive about pursuing partnership working, and their priority was staff training to enable frontline workers to support tenants at end of life.^30^ The report concludes that housing providers can play a key part in delivering effective, person-centred end-of-life care within communities and makes recommendations aimed at both improving support for individuals and system-level changes (e.g. that local authorities should involve housing providers in the integrated planning of community-based approaches to meeting end-of-life needs). The study did not, however, include the perspectives of tenants and the authors also acknowledged that conversations tended to focus on sheltered accommodation and extra care schemes.

What our study adds to this limited body of research is the perspective of a broader range of adults affected by dying or bereavement and living on a low income in social or privately rented housing. While recognising the challenges to capacity that services supporting people at end of life already face, our findings suggest some specific ways that both housing and healthcare providers could improve the experience of those they support.

For housing providers these include the following:

- An holistic approach to assessing the housing needs of someone with a life-limiting illness, addressing not only their physical concerns such as mobility, accessibility, and heating, but social needs including proximity and access to existing support networks.
- Proactively addressing the potential needs of those co-habiting and encouraging them to talk openly about their needs as carers and any concerns about tenancy and future housing status.
- Bereavement policies that signpost to bereavement support offering practical as well as emotional support and potentially longer-term support/advocacy from a trusted individual.
- A compassionate and sensitive approach to relatives with responsibility for clearing a deceased person’s home.

The data also suggest two ways in which healthcare providers could make a difference.

- Early identification of housing issues or needs (affecting the patient and their family).

Having ‘permission’ to talk about housing and financial worries is important both for patients and their families, and as the professionals most likely to have contact with patients with life-limiting illnesses, healthcare practitioners are well placed to enable patients and carers to express anxieties, perhaps as part of an advance care planning process. The advice workers who participated in this study were employed either by hospices or charities such as Macmillan, and of course not all palliative care practitioners are able to signpost to this kind of support as it is not universally available. This underlines the need for an integrated system as described above that can assess and respond to health, housing, and welfare needs, and include those of the family as well as the patient.

- Training for healthcare practitioners in understanding and responding to social determinants of health.

Again, while recognising that healthcare practitioners may not have the capacity to respond to wider needs, they may be the only services with which a patient has contact. As Stadjuhar *et al.*^23^ found, people in insecure housing are more likely to engage with end-of-life care provision when healthcare professionals understand and demonstrate awareness of the social determinants of health. This means that those involved in supporting people at end of life need to sensitively seek to understand an individual’s circumstances, be aware of the impact of inequalities across the lifecourse, and recognise the importance of building trust to counter potential fears.

Interestingly, experiences during the COVID-19 pandemic have prompted some recent discussion in the field about a need for trauma-informed palliative care that recognises the psychological impact of terminal illness.^31^ The principles of trauma-informed care developed in other fields such as social care (e.g. the centrality of safety and trustworthiness) may also be useful for helping practitioners across sectors understand the impact of inequalities and trauma across the lifecourse on attitudes towards, and access to, end-of-life care.

In a critique of the palliative care curriculum, Abel and Kellehear highlight the absence of public health approaches to care and support at the end of life.^32^ Their analysis of the curriculum shows that the ‘psychosocial’ aspects of dying focus on the psychological concerns of the individual, with no recognition of social issues that may impact on the patient and their family, for example, workplace policies or poor partnerships between health services, social care providers, and communities. While healthcare providers cannot solve housing issues for their patients, an awareness of their potential impact at end of life may help them refer families to appropriate support at an earlier stage and prompt a greater recognition within the palliative care field that housing providers and policy-makers are key partners within a collaborative public health approach to building more compassionate ‘death systems’.

## Conclusion

We recognise the limitations of this study in that it is an in-depth qualitative study with a relatively small sample size, and the bereaved individuals who participated in this study were mostly recruited through a professional they had been in contact with or through our relations with community networks. As a result, most participants had received support in some form either before or after their bereavement, and there is less data related to the experiences of those who did not access support. However, the fact that issues related to housing emerged so strongly from an enquiry into what is difficult, and what is important for individuals on a low income at end of life or in bereavement, suggests that housing needs to be recognised as an important element within public health approaches to palliative care. While the points above mainly focus on actions for healthcare and housing providers, they suggest potentially huge benefits to cross-sector learning and collaboration. Marie Curie’s report^30^ noted that most of their recommendations for housing providers are not resource intensive and may lead to savings elsewhere in the system. The introduction of Integrated Care Systems across the United Kingdom seems an ideal opportunity to explore the potential for housing providers to make a valuable contribution to addressing the inequalities inherent with current ‘death systems’.
